# The Adaptations of *E. coli* SM10λpir (pUCP24T) Under Constant Sub‐MIC Gentamicin Treatment

**DOI:** 10.1155/cjid/6978370

**Published:** 2026-05-12

**Authors:** Yuting He, Guoxiu Xiang, Guosheng Zhong, Jianming Zeng, Cha Chen, Bin Huang

**Affiliations:** ^1^ Department of Laboratory Medicine, The First Affiliated Hospital, Sun Yat-sen University, Guangzhou, 510080, China, sysu.edu.cn; ^2^ Department of Laboratory Medicine, The Sixth Affiliated Hospital, Sun Yat-sen University, Guangzhou, China, sysu.edu.cn; ^3^ Department of Laboratory Medicine, Guangdong Provincial Hospital of Traditional Chinese Medicine, Guangzhou, Guangdong, China, gdhtcm.com

**Keywords:** bacterial adaptation, fitness cost, horizontal gene transfer, plasmid copy number, sub-MIC gentamicin, whole genome sequencing

## Abstract

**Background:**

Antibiotics, as a selection stress, could trigger specific responses in bacterial pathogens. This study aimed to investigate adaptive changes of *E. coli* SM10λpir (pUCP24T) under constant treatment of sub‐MIC Gm (gentamicin).

**Methods:**

*E. coli* SM10λpir (pUCP24T) underwent continuous passage culture by serial transfer for 50 days on agar plates containing 30 μg/mL Gm to obtain *E. coli* SM10λpir (pUCP24T)‐E. Two strains were compared for the horizontal gene transfer ability, stability of plasmid pUCP24T, fitness cost, and expression of conjugation‐related genes. Based on whole genome and RNA sequencing data, functional enrichment analysis (GO and KEGG) was conducted, along with analyses of plasmid sequencing depth, SNPs, and differentially expressed genes (DEGs).

**Results:**

The conjugation frequency of *E. coli* SM10λpir (pUCP24T)‐E with recipient PAO1 was higher, and its *traI* expression was significantly upregulated (*p* < 0.05). In the same strain, the growth rate and competition index were lower (*p* < 0.05); the sequencing depth of plasmid pUCP24T and the relative expression of the *rep* gene were much higher (*p* < 0.05), but the plasmid showed reduced stability. Functional enrichment analysis suggested a possible enhancement of certain physiological processes and metabolic pathways. A total of 1294 DEGs were detected, with obvious upregulation of *hycB*, *hycD*, *nikE*, *cspA*, and *nanA*, and obvious downregulation of *gadB*, *gadC*, *yeiQ*, and *yjiH*, transcription factors (*appY*, *gadE*), and sRNAs (*arrS*, *isrC*). Additionally, the expression of aerobic respiratory pathway genes (*cyoABCDE*) in *E. coli* SM10λpir (pUCP24T)‐E increased significantly (*p* < 0.05).

**Conclusions:**

The enhanced conjugation frequency during adaptation may be attributed to increased expression of the transfer gene *traI* and an elevated copy number of plasmid pUCP24T. A heavier fitness cost was imposed on the host during this process. Aerobic respiration and metabolic efficiency were likely potentiated. sRNA *isrC* was hypothesized to inhibit aerobic respiration by targeting the cytochrome bo oxidase subunit *cyoD*.

## 1. Introduction

To successfully survive in changing environments, bacteria must adapt to multiple stress factors. These factors encompass changes in temperature, pH, antibiotic concentration, aerobic and anaerobic environments, ion concentration, and nutrition [1, 2]. Horizontal gene transfer (HGT) involves the sharing of genetic material between organisms that are not in a parent–offspring relationship. HGT has been extensively recognized as a mechanism for bacterial adaptation [3]. HGT through plasmids plays a crucial role in the transfer of antimicrobial resistance. Plasmids are vital for bacterial adaptation to novel environments by providing various accessory genes that encode functional traits and accelerate the adaptation of bacteria to environmental stressors. Thus, the evolution of plasmid‐encoded genes can be promoted, and the adaptation of their bacterial hosts can be facilitated [4]. Antibiotics, as a selection stress, can trigger specific responses in bacterial pathogens, leading to mutational adaptations, acquisition of genetic material, or alteration of gene expression. The carriage of plasmids is of critical significance in bacterial evolution and rapid adaptation. Accordingly, the bacterial host is enabled to survive under antibiotic pressure, whereas a fitness cost is imposed on the host [5]. The fate of plasmid‐bearing strains will be determined by the fitness cost during adaptation. Bacteria are well known for their extremely high adaptability in stressful environments, and bacterial populations can adopt different strategies to adapt to antibiotic treatment, such as *de novo* mutations, HGT [6]. Moreover, antibiotics typically induce major physiological changes in bacteria [7]. Recently, many studies have focused on the adaptation of bacteria and plasmids under antibiotic treatment, including genetic mutations, gene expression changes, and changes in physiological processes. In this work, we investigated the bacterial and plasmid adaptive changes under constant sub‐minimum inhibitory concentration (sub‐MIC) Gm (gentamicin) treatment using the *E. coli* SM10λpir (pUCP24T) strain. *E. coli* SM10λpir (pUCP24T) was obtained when plasmid pUCP24T, which carries the *aacC1* gene conferring resistance to Gm, was transferred to *E. coli* SM10λpir. By transferring a single *E. coli* SM10λpir (pUCP24T) colony from an LB plate containing 30 μg/mL Gm to a new LB plate containing 30 μg/mL Gm daily for 50 days (a total of 50 transfers), the adaptive changes undergone by the plasmid and host were investigated, including HGT ability, plasmid stability and copy number, fitness cost, and the expression of conjugation‐related genes. Moreover, whole genome sequencing and RNA sequencing were performed to explore the possible mechanisms behind the adaptations, providing new insights on adaptations of bacteria and plasmids.

## 2. Materials and Methods

### 2.1. Bacterial Strains and Characteristics

The recombinant strain *E. coli* SM10λpir (pUCP24T) was created by transferring the plasmid pUCP24T into *E. coli* SM10λpir through chemical transformation. The employed plasmid pUCP24T is a conjugative plasmid constructed from the pUCP24 plasmid with an inserted *oriT* sequence [8]. The *oriT* sequence is the conjugative reaction initiation sequence of pCVD442. The pUCP24T carries the *aacC1* gene, which confers resistance to Gm. The MIC of *E. coli* SM10λpir (pUCP24T) to Gm was determined to be 2048 μg/mL. The strain *Pseudomonas aeruginosa* PAO1, which is resistant to ampicillin (Amp) and sensitive to Gm, served as the recipient in the conjugation experiments.

### 2.2. Serial Transfer Under Constant Gm Treatment

*E. coli* SM10λpir (pUCP24T) was cultured on LB agar plates containing 30 μg/mL Gm using quadrant streaking. A single colony was randomly selected from the plate and transferred to a new LB agar plate containing 30 μg/mL Gm. This plate was maintained at 37°C for 24 h using quadrant streaking. This process was repeated daily for 50 days. Finally, the resulting *E. coli* SM10λpir (pUCP24T) underwent continuous passage culture by serial transfer for 50 days on agar plates containing 30 μg/mL Gm. The new strain was designated as *E. coli* SM10λpir (pUCP24T)‐E.

### 2.3. Conjugation and Expression of Conjugation‐Related Genes

*E. coli* SM10λpir (pUCP24T) and *E. coli* SM10λpir (pUCP24T)‐E served as the donors. *P. aeruginosa* PAO1 was the recipient. Conjugation experiments were performed to explore the transfer capability of pUCP24T plasmid [9]. Equal quantities (1 × 10^7^ CFU/mL) of mid‐logarithmic phase donor and recipient cells were cocultured in 200 μL of LB broth in 96‐well plates. After a 6‐h mating at 37°C, 20 μL of the mixed cultures was spread on LB agar containing 30 μg/mL Gm plus 100 μg/mL Amp. The conjugation frequency was calculated by dividing the number of transconjugants by the number of donors. These experiments were replicated three times. Bacterial counts were performed using the Sysmex UF‐1000i Automated Urine Particle Analyzer (Tokyo, Japan). Cultures of *E. coli* SM10λpir (pUCP24T) and *E. coli* SM10λpir (pUCP24T)‐E were grown in 3 mL of LB broth supplemented with 30 μg/mL Gm, with shaking at 200 rpm at 37°C. At the mid‐logarithmic phase, 1.5‐mL *E. coli* SM10λpir (pUCP24T) and *E. coli* SM10λpir (pUCP24T)‐E cultures were collected, respectively, for RNA extraction. Total RNA was isolated using RNAiso Plus reagent (TaKaRa, Japan). The purity of RNA was assessed using a NanoDrop 2000 (Thermo, USA). Reverse transcription of 1 μg of total RNA was conducted using the PrimeScript RT reagent kit with gDNA Eraser (TaKaRa, Japan). The expression of conjugation‐related genes (*traI*, *traJ*) and global regulatory genes (*korA*, *korB*) was analyzed by SYBR green‐based quantitative reverse transcription PCR (RT‐qPCR). The qPCR was performed on a ViiA 7 Dx system (Applied Biosystems, USA) using SYBR green qPCR master mixes (TaKaRa, Japan). The gene *rpoD* served as the reference gene. All the primers were validated for efficiency and are listed in Table S1.

### 2.4. The Stability of Plasmid pUCP24T

*E. coli* SM10λpir (pUCP24T) and *E. coli* SM10λpir (pUCP24T)‐E strains were cultured in 4 mL of LB broth containing 30 μg/mL Gm with shaking at 200 rpm at 37°C overnight. The following day, 40 μL of these overnight cultures was transferred to 4 mL of fresh LB broth without antibiotics and further cultured under the same conditions. This process was repeated every 24 h. Both strains were propagated through 1% serial transfer at 24‐h intervals. At the 12‐h time point, when the strains reached the logarithmic growth phase with OD_600_ between 0.6 and 0.8, 50 μL of the cultures were taken, diluted, and spread on LB agar containing 30 μg/mL Gm and on LB agar without antibiotics. Then, the procedure was repeated every 24 h. The stability of plasmid pUCP24T was assessed by calculating the ratio of the number of colonies on plates containing Gm to the number of colonies on antibiotic‐free plates. Colonies on the plates containing 30 μg/mL Gm and on the plates without Gm were selected randomly. Each colony was suspended in 20 μL sterile water, boiled for 10 min, and centrifuged to get the supernatant as the templates. Primers targeting the *oriT* conserved region sequence of plasmid pUCP24T were used to conduct the PCR amplification. The PCR products underwent agarose gel electrophoresis. The appearance of the target band indicated the presence of pUCP24T, while its disappearance indicated the loss of pUCP24T.

### 2.5. Fitness Cost

#### 2.5.1. Growth Rate

The *E. coli* SM10λpir (pUCP24T) and *E. coli* SM10λpir (pUCP24T)‐E were cultured in LB broth supplemented with 30 μg/mL Gm at 37°C with shaking overnight. Concurrently, the control strain, *E. coli* SM10λpir, was cultured under identical conditions. The next day, 50 μL of the overnight cultures from each of the three strains was inoculated into test tubes containing 5 mL of fresh LB broth and cultured at 37°C with shaking at 200 rpm. At hourly intervals, 200 μL samples from each strain were transferred to a 96‐well plate for OD_600_. The OD_600_ values were measured using a Synergy 2 multifunctional microplate reader (BioTek, USA). Growth curves were subsequently plotted with time on the abscissa and OD_600_ values on the ordinate.

#### 2.5.2. Pairwise Competition

To assess the relative fitness of *E. coli* SM10λpir (pUCP24T) and *E. coli* SM10λpir (pUCP24T)‐E, both strains were cocultured with *E. coli* SM10λpir in LB broth without antibiotics at a ratio of 1:1 to perform a competition experiment. *E. coli* SM10λpir (pUCP24T), *E. coli* SM10λpir (pUCP24T)‐E, and *E. coli* SM10λpir exhibited exponential growth at 37°C in LB broth supplemented with 30 μg/mL Gm. One mL cultures of *E. coli* SM10λpir (pUCP24T) and *E. coli* SM10λpir (pUCP24T)‐E were transferred to separate EP tubes and centrifuged immediately. The supernatant was discarded, and cell pellets were resuspended in 1 mL of LB broth without antibiotics. This washing procedure was repeated three times. Subsequently, the bacterial concentration was determined. Cultures containing 5 × 10^4^ CFU of *E. coli* SM10λpir (pUCP24T) and 5 × 10^4^ CFU of *E. coli* SM10λpir (pUCP24T)‐E were mixed with 5 × 10^4^ CFU of *E. coli* SM10λpir in 1 mL of LB broth, respectively. These mixed cultures were incubated for 12 h at 37°C without shaking. After 12 h, 50 μL of each culture was plated on LB agar plates supplemented with 30 μg/mL Gm and on LB agar plates without antibiotics. The competition experiment was performed in triplicate. Relative fitness was expressed as the competition index (CI), calculated as the ratio of the mean CFU between the resistant and susceptible strains at t_12_ divided by the same ratio at t_0_ [10]. The CFU of the resistant strains was represented as the CFU on the plates containing 30 μg/mL Gm. The CFU of the susceptible strains was determined by subtracting the CFU on the plates containing 30 μg/mL Gm from the CFU on the plates without antibiotics.

### 2.6. The Whole Genome Sequencing and RNA Sequencing

Strains of *E. coli* SM10λpir (pUCP24T) and *E. coli* SM10λpir (pUCP24T)‐E were cultured at 37°C with shaking in LB broth containing 30 μg/mL Gm overnight. The following day, 100 μL of these cultures was inoculated into 10 mL of LB broth supplemented with 30 μg/mL Gm and incubated at 37°C with shaking until reaching the logarithmic growth phase. The cultures were then collected for whole genome sequencing on the Illumina NextSeq 500 platform. The paired‐end raw reads were filtered using Fastp v0.12.5. *De novo* assembly and annotation were conducted using Unicycler v0.4.9b and Prokka v1.14.6. Breseq v0.35.5 was employed to analyze the single‐nucleotide variants (SNPs) between *E. coli* SM10λpir (pUCP24T) and *E. coli* SM10λpir (pUCP24T)‐E. Concurrently, RNA sequencing was performed on the Illumina HiSeq 2000 platform. Each strain was sequenced only once. The DNA extraction, library construction, sequencing, and analysis were all performed in the same batch, minimizing any batch effect. The sequencing data volume was 1 Gb, and the depth of sequencing was 25X. The Q30 of the original sequencing data was above 90%. The raw reads were processed using Fastp v0.12.5, and sequences were aligned to the reference genomes of *E. coli* MG1655 K‐12 (GenBank accession NC_000913.3), RK2 (GenBank accession NC_001621.1), and pUCP24T (GenBank accession MF098686.1) using HISAT2. Quantification was performed with StringTie. The original count was normalized to fragment per kilobase million (FPKM) using the edgeR software package for statistical analysis. The online website DAVID (https://david.ncifcrf.gov/tools.jsp) was employed to perform the enrichment of differentially expressed genes (DEGs) for GO function analysis. The enrichment of DEGs was performed using the online tool KOBAS (https://kobas.cbi.pku.edu.cn/kobas3) for KEGG function analysis. The Sangerbox online website (https://vip.sangerbox.com/home.html) was used for data visualization. The protein interaction network of DEGs was analyzed through Cytoscape v3.9.0.

### 2.7. Plasmid Copy Number Analysis

Plasmid copy numbers were determined as described previously [11]. SYBR Green‐based quantitative PCR (qPCR) was used to assess the copy numbers of the pUCP24T plasmid. Genomic DNA was extracted using the MiniBEST Bacteria Genomic DNA Extraction Kit (TaKaRa, Japan) and treated with the restriction endonuclease *Hin*dIII (TaKaRa, Japan) to reduce the quantitative deviations associated with topology. The qPCR was performed on the ViiA 7 Dx system (Applied Biosystems, USA) using SYBR green qPCR master mixes (TaKaRa, Japan). The copy number was determined by quantifying a 195‐bp segment within the *rep* gene of pUCP24T and comparing it to a 157‐bp segment within the chromosomal gene *atpG*, which served as an internal control. The following primers were used: *rep*‐F: 5′‐TTC​ACC​AAA​GAC​ATG​CTG​CC‐3′; *rep*‐R: 5′‐ATA​CCG​GCC​TTC​CAG​TTG​AA‐3′; *atpG*‐F: 5′‐GTC​GGT​CCA​GGT​CTT​CAT​TT‐3′; *atpG*‐R: 5′‐TGC​ACA​CGG​TAA​TCT​GGA​AT‐3′. The plasmid copy number was calculated based on the ratio of the copy numbers of the *rep* and *atpG* genes.

### 2.8. Statistical Analysis

Student’s *t*‐test, analysis of variance (ANOVA), and the Wilcoxon test were used for comparisons between groups according to the data type and sample size. All results with a *p* value less than 0.05 were considered significant. The false discovery rate (FDR) and fold change (FC) were employed to identify DEGs. Genes were considered differentially expressed only if FDR < 0.05 and |logFC| > 1.

## 3. Results

### 3.1. Increased Conjugation Efficiency With the Recipient PAO1 and Expression of traI

After serial transfers on LB agar plates containing 30 μg/mL Gm daily for 50 days, *E. coli* SM10λpir (pUCP24T)‐E was isolated on antibiotic‐containing plates. Both *E. coli* SM10λpir (pUCP24T) and *E. coli* SM10λpir (pUCP24T)‐E were used to conduct conjugation with the recipient PAO1. The conjugation frequency is shown in Figure 1(a). Compared with *E. coli* SM10λpir (pUCP24T), the conjugation frequency of *E. coli* SM10λpir (pUCP24T)‐E with the recipient PAO1 was significantly higher (*p* < 0.05). To further explore the possible molecular mechanisms underlying the increased conjugative efficiency, the expression of conjugation‐related genes was analyzed. These included the major global regulatory genes (*korA* and *korB*), which influence plasmid replication, and the conjugation‐related genes *traI* (encoding the conjugative transfer relaxase) and *traJ* (activating *tra* gene expression). Compared with *E. coli* SM10λpir (pUCP24T), *traI* expression in *E. coli* SM10λpir (pUCP24T)‐E was significantly higher (*p* < 0.05), while *traJ*, *korA,* and *korB* showed no significant differences between the two strains (*p* > 0.05) (Figure 1(b)).

FIGURE 1Conjugation and expression of conjugation‐related genes. (a) *E. coli* SM10λpir (pUCP24T) and *E. coli* SM10λpir (pUCP24T)‐E were taken as donors, and the *P. aeruginosa* PAO1 was used as the recipient. The conjugation frequency of each strain with *P. aeruginosa* PAO1 was determined. Compared with *E. coli* SM10λpir (pUCP24T), the conjugation frequency of *E. coli* SM10λpir (pUCP24T)‐E with recipient PAO1 was significantly higher (*p* < 0.05). (b) The expression of conjugation‐related genes between *E. coli* SM10λpir (pUCP24T) and *E. coli* SM10λpir (pUCP24T)‐E was compared. Compared with *E. coli* SM10λpir (pUCP24T), *traI* expression in *E. coli* SM10λpir (pUCP24T)‐E was significantly higher (*p* < 0.05), while *traJ*, *korA*, and *korB* showed no significant differences between the two strains (*p* > 0.05). These experiments were replicated three times.

**Figure:**
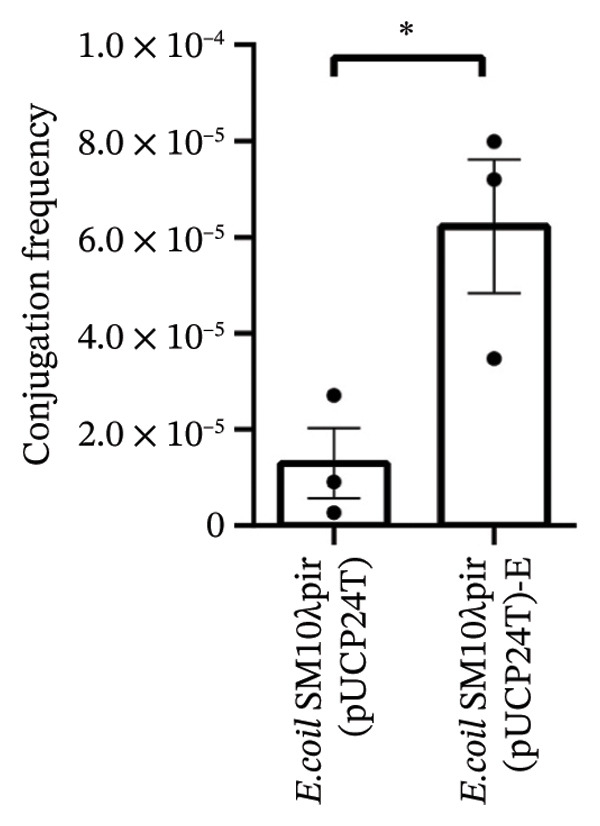
(a)

**Figure:**
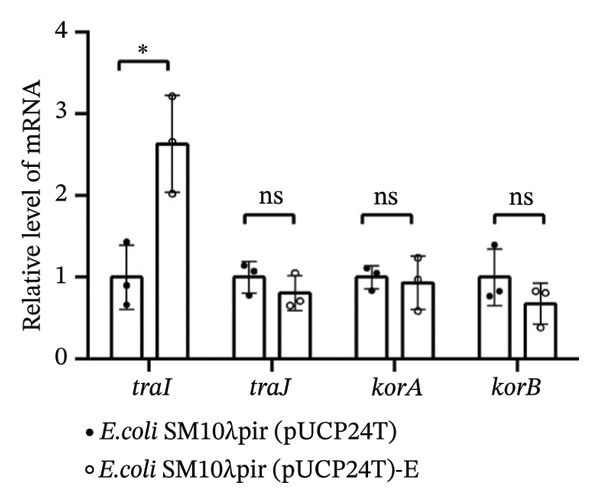
(b)

### 3.2. The Instability of Plasmid pUCP24T

Every 24 h, the stability of plasmid pUCP24T carried out by the two strains was calculated (Figure 2(a)). The plasmid‐carrying rates for the *E. coli* SM10λpir (pUCP24T) and *E. coli* SM10λpir (pUCP24T)‐E ranged from 0.69 to 0.74 and 0.63 to 0.66, respectively, until 60 h, indicating that the stability of plasmid pUCP24T in both strains was relatively stable until 60 h. Subsequently, the plasmid‐carrying rate of pUCP24T in *E. coli* SM10λpir (pUCP24T)‐E dropped from 0.69 at 60 h to 0.42 at 84 h. The plasmid pUCP24T in *E. coli* SM10λpir (pUCP24T)‐E nearly disappeared at 108 h and was absent at 180 h, remaining absent until 228 h. Meanwhile, the plasmid‐carrying rate of pUCP24T in *E. coli* SM10λpir (pUCP24T) dropped from 0.69 at 60 h to 0.53 at 132 h and ranged from 0.53 to 0.44 from 132 h to 228 h. In an environment free of Gm, the plasmid pUCP24T in *E. coli* SM10λpir (pUCP24T)‐E showed greater instability compared to *E. coli* SM10λpir (pUCP24T). After serial transfers on Gm‐containing agar plates for 50 days, the stability of pUCP24T tended to decrease (Figure 2(a)).

FIGURE 2The stability and fitness cost of plasmid pUCP24T. (a) The plasmid‐carrying rate of *E. coli* SM10λpir (pUCP24T) and *E. coli* SM10λpir (pUCP24T)‐E in LB broth without antibiotic was measured over 228 h. The plasmid‐carrying rates for *E. coli* SM10λpir (pUCP24T) and *E. coli* SM10λpir (pUCP24T)‐E ranged from 0.69 to 0.74 and 0.63 to 0.66, respectively, during the first 60 h. Subsequently, the plasmid‐carrying rate of pUCP24T in *E. coli* SM10λpir (pUCP24T)‐E dropped from 0.69 at 60 h to 0.42 at 84 h. It nearly disappeared by 108 h and was absent at 180 h, remaining undetectable until 228 h. Meanwhile, the plasmid‐carrying rate of pUCP24T in *E. coli* SM10λpir (pUCP24T) dropped from 0.69 at 60 h to 0.53 at 132 h, and then ranged from 0.53 to 0.44 between 132 h and 228 h. (b) The growth curves of *E. coli* SM10λpir (pUCP24T), *E. coli* SM10λpir (pUCP24T)‐E, and *E. coli* SM10λpir were measured. Among these strains, *E. coli* SM10λpir exhibited the fastest growth. The growth rates of both plasmid‐carrying strains were reduced, with *E. coli* SM10λpir (pUCP24T)‐E showing a significantly lower growth rate compared with *E. coli* SM10λpir (pUCP24T) (*p* < 0.05). (c) The competition index (CI) of *E. coli* SM10λpir (pUCP24T) and *E. coli* SM10λpir (pUCP24T)‐E was determined relative to *E. coli* SM10λpir. The mean CIs for *E. coli* SM10λpir (pUCP24T) and *E. coli* SM10λpir (pUCP24T)‐E were 0.52 and 0.30, respectively, both of which were less than 1. Furthermore, the CI for *E. coli* SM10λpir (pUCP24T)‐E was significantly lower than that for *E. coli* SM10λpir (pUCP24T) (*p* < 0.05). These experiments were replicated three times.

**Figure:**
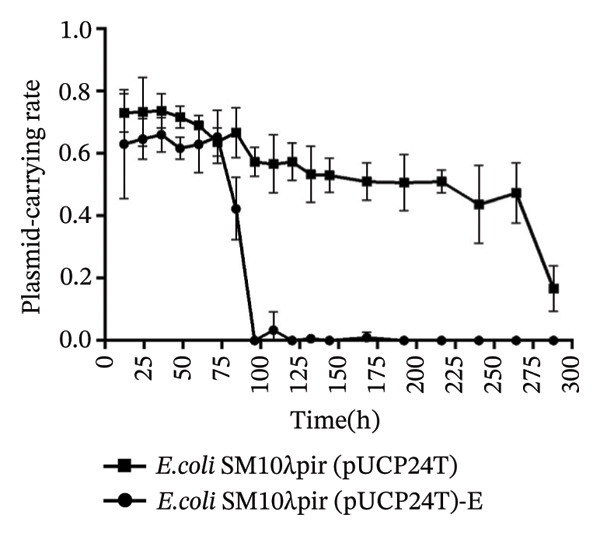
(a)

**Figure:**
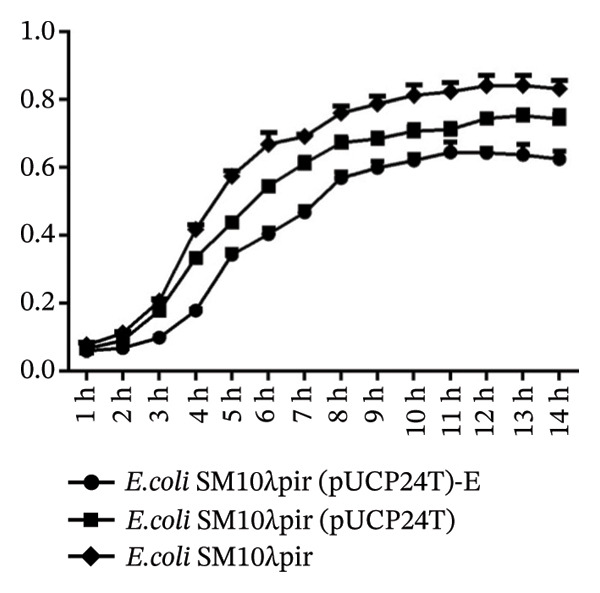
(b)

**Figure:**
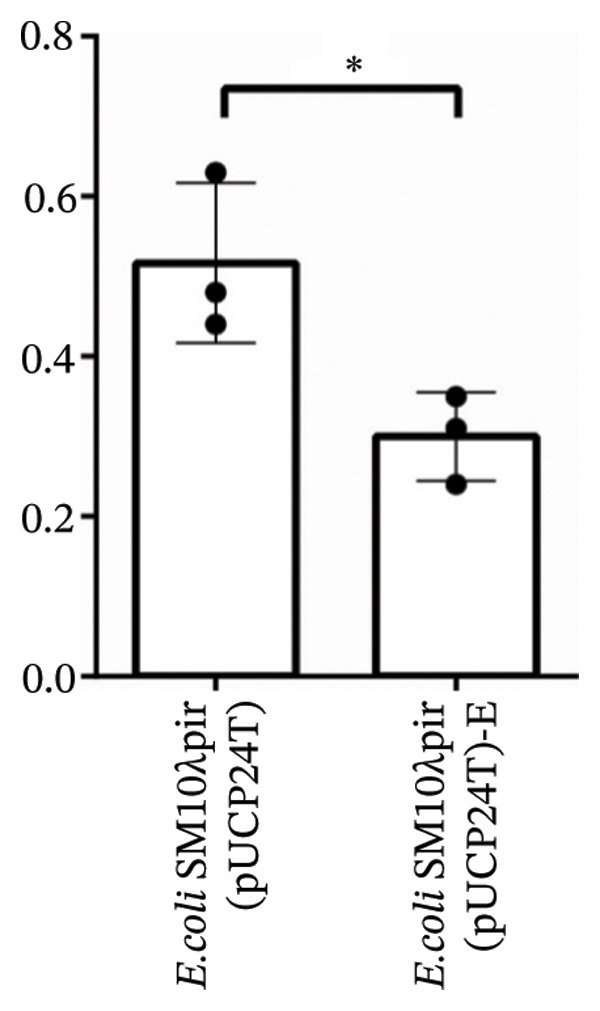
(c)

### 3.3. Lower Growth Rate and Competitive Inferiority

To evaluate the fitness cost associated with carrying pUCP24T plasmids, we observed the growth rates of the three strains and calculated their competition indices. Among these strains, *E. coli* SM10λpir exhibited the fastest growth. Compared with *E. coli* SM10λpir (pUCP24T), the growth rate of *E. coli* SM10λpir (pUCP24T)‐E was significantly lower (*p* < 0.05) (Figure 2(b)). In pairwise competitions, the mean CIs for *E. coli* SM10λpir (pUCP24T) and *E. coli* SM10λpir (pUCP24T)‐E were 0.52 and 0.30, respectively, both of which were less than 1. Furthermore, the CI for *E. coli* SM10λpir (pUCP24T)‐E was significantly lower than that for *E. coli* SM10λpir (pUCP24T) (*p* < 0.05). The slower growth rate and competitive inferiority of *E. coli* SM10λpir (pUCP24T)‐E suggested that the serial transfer on sub‐MIC Gm agar plates could incur a heavier fitness cost on the strain.

### 3.4. Increased pUCP24T Copy Number and Expression Analysis of a Series of Physiological Process‐Related Genes Including Respiratory Enzyme Genes

**FIGURE 3:**
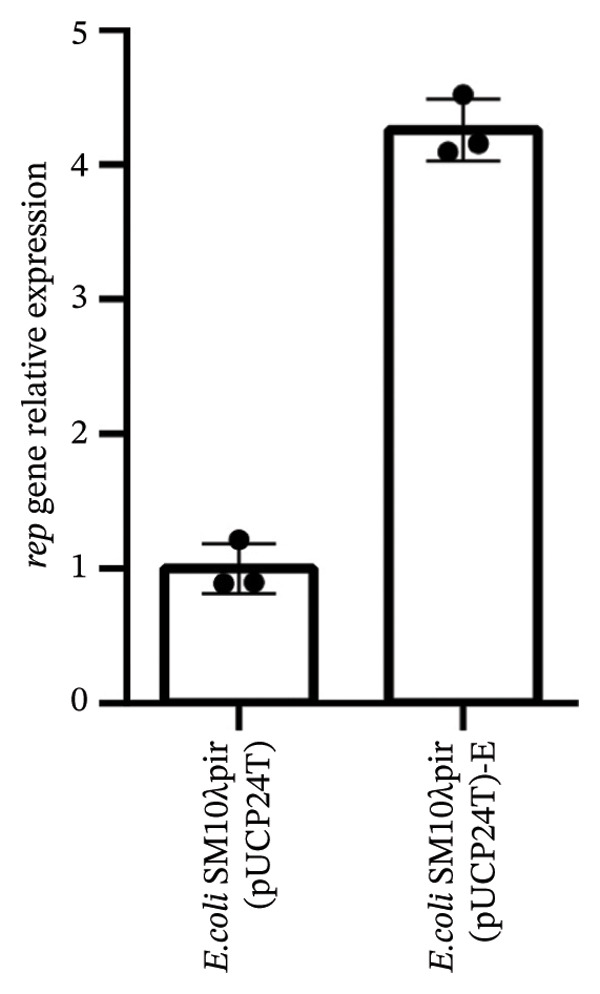
Plasmid pUCP24T copy number analysis. The *rep* gene relative expression represented the plasmid copy calculated as the ratio of the copy numbers of *rep* and *atpG*. The relative expression of the *rep* gene in *E. coli* SM10λpir (pUCP24T)‐E was much higher than that in *E. coli* SM10λpir (pUCP24T) (*p* < 0.05). These experiments were replicated three times.

The original sequence data from *E. coli* SM10λpir (pUCP24T)‐E were aligned with those of the ancestral strain *E. coli* SM10λpir (pUCP24T) using Breseq v0.35.5. The mutations in *E. coli* SM10λpir (pUCP24T)‐E primarily occurred in the e14 phage of SM10λpir, most of which were synonymous, with only three missense mutations: *icd* L375M (TTA ⟶ CTG), *icd* L375M (TTA ⟶ CTG), and *pinE* T98S (ACA ⟶ TCT). Two mutations occurred in the intergenic regions between *rep* and *aacC1* of the pUCP24T plasmid (Table S2). Regarding the plasmid pUCP24T sequencing depth in the two strains, the average sequencing depth of pUCP24T in *E. coli* SM10λpir (pUCP24T)‐E was approximately five times higher than that in *E. coli* SM10λpir (pUCP24T), indicating an increase in pUCP24T copy number. Moreover, the copy number of plasmid pUCP24T relative to the chromosome was also determined by qPCR of *rep* relative to the chromosomal *atpG* gene. The relative expression of the *rep* gene in *E. coli* SM10λpir (pUCP24T)‐E was much higher than that in *E. coli* SM10λpir (pUCP24T) (*p* < 0.05), consistent with the whole genome sequencing results (Figure 3).

Compared with *E. coli* SM10λpir (pUCP24T), *E. coli* SM10λpir (pUCP24T)‐E exhibited a total of 1294 DEGs. Of these, 1003 genes were upregulated and 291 genes were downregulated, indicating significant alterations in gene expression during the adaptation. Among the DEGs, the genes that were relatively prominently upregulated included those encoding formate dehydrogenase (*hycB*, *hycD*), Ni^2+^ uptake protein (*nikE*, *nikC*), *N*‐acetylneuraminic lyase (*nanA*), and cold shock protein A (*cspA*). Conversely, genes associated with the glutamate‐dependent acid resistance system 2 (*gadB*, *gadC*), a hypothesized NAD‐dependent dehydrogenase (*yeiQ*), a gate family protein (*yjiH*), and the self‐recognized antigen Ag43 (*flu*) were substantially downregulated. The expression of *isrC*, a small noncoding RNA (sRNA) with unknown function, and sRNA *arrS*, which regulates the bacterial acid stress response, were significantly downregulated (Figure 4(a)). Functional enrichment analysis using GO and KEGG revealed that the expression of genes related to several physiological processes and metabolic pathways was upregulated. The physiological processes and metabolic pathways included ribosome synthesis, translation, antigen biosynthesis, exopolysaccharide biosynthesis, cytochrome oxidase activity, and proton transport ATP synthase activity, as well as the tricarboxylic acid (TCA) cycle, glycolysis/gluconeogenesis, pyruvate metabolism, purine metabolism, propionic acid metabolism, amino sugar and nucleotide sugar metabolism, and lipopolysaccharide biosynthesis. In contrast, the expression of genes related to physiological processes such as DNA integration, translocation, and bacterial chemotaxis was downregulated (Figures 4(b) and 4(d)). The DEGs were submitted to the STRING database online for analysis and construction of the interaction network. The interaction data were imported into Cytoscape v3.9.0 software, and the GO database was used for pathway analysis and functional study. Functional annotation revealed that the DEGs were mainly linked to the ribosome, cytosolic ribosome, intracellular non–membrane‐bounded organelle, ribosomal subunit, non–membrane‐bounded organelle, and cytosolic large ribosomal subunit (Figure 4(c)). Compared with *E. coli* SM10λpir (pUCP24T), the expression levels of key aerobic respiration enzyme genes, including ATP synthase (*atpADGH*), succinate dehydrogenase (*sdhAB*), and particularly the cytochrome bo oxidase (*cyoABCDE*), were upregulated in *E. coli* SM10λpir (pUCP24T)‐E (Figure 4(e)).

FIGURE 4The whole genome sequencing and RNA sequencing analysis. (a) Volcano plot of DEGs. The abscissa represents −log10 (FDR), and the ordinate represents log2 fold change (FC). |log2FC| > 1 was used as the cutoff for defining DEGs. Red indicates upregulation, and green indicates downregulation. Yellow boxes represent transcription factors, and purple boxes represent sRNAs. (b) Bubble plot of GO functional enrichment analysis. The ordinate represents the GO term, and the abscissa represents the gene ratio. Bubble size indicates the number of enriched genes, bubble color indicates the *p* value, and bubble shape indicates the direction of enrichment. (c) Protein–protein interaction network of DEGs. Color shade indicates the FDR value. Blue indicates downregulation, red indicates upregulation, and the colored circles outside indicate GO functional enrichment. The image was generated using Cytoscape v3.9.0. (d) Bubble plot of KEGG functional enrichment analysis. The ordinate represents the KEGG pathway, and the abscissa represents the rich factor. Bubble size indicates the number of enriched genes, bubble color indicates the *p* value, and bubble shape indicates the direction of enrichment. (e) Expression changes of genes related to energy metabolism. Color shade indicates the magnitude of log2FC.

**Figure:**
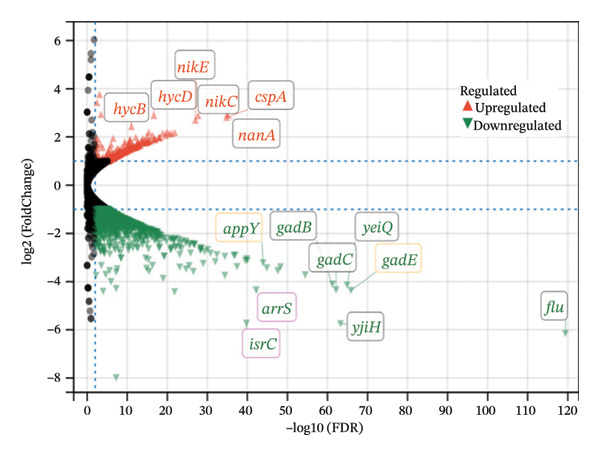
(a)

**Figure:**
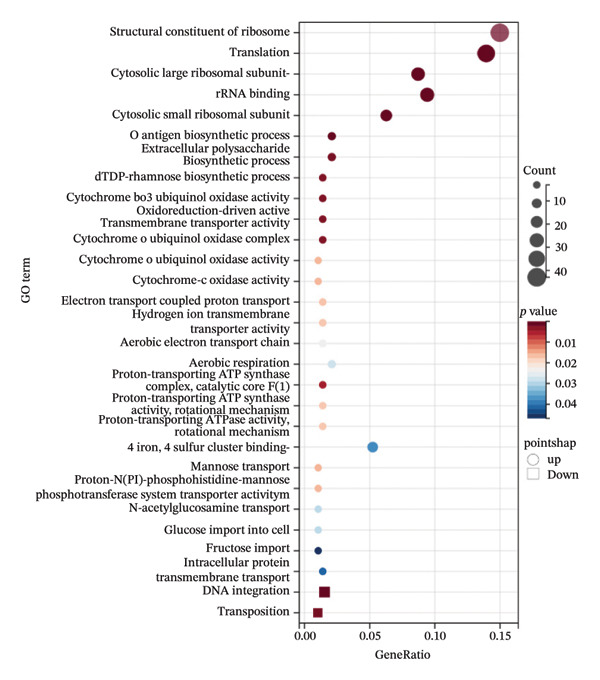
(b)

**Figure:**
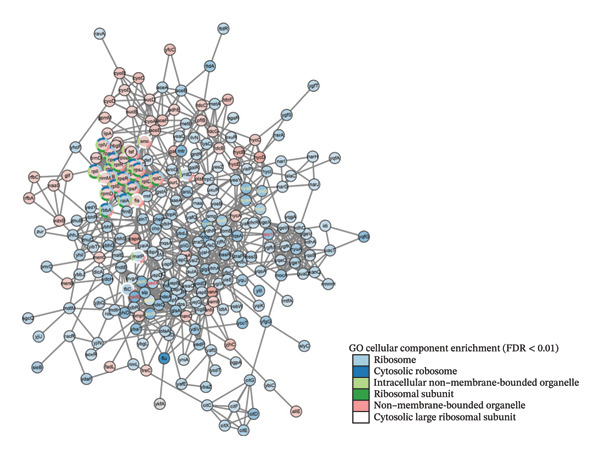
(c)

**Figure:**
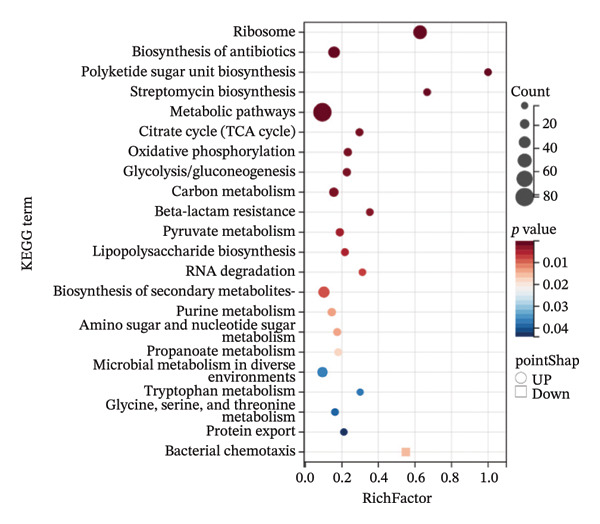
(d)

**Figure:**
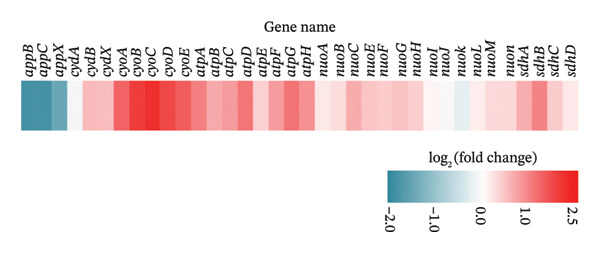
(e)

## 4. Discussion

Antibiotics exert selection pressure for antibiotic resistance, even at low concentrations, facilitating the spread of antibiotic resistance genes among different bacterial species [12]. HGT is a prevalent event in the microbial ecosystem, recognized as a mechanism of adaptation in bacteria [13]. Exposure to sub‐MIC levels of antibiotics can lead to various effects, including the induction of virulence and HGT [14]. In our study, the MIC of *E. coli* SM10λpir (pUCP24T) for Gm is 2048 and 30 μg/mL is approximately equivalent to 1/50 of the MIC. *E. coli* SM10λpir (pUCP24T) was often recovered on LB agar plates containing 30 μg/mL Gm in our laboratory, and the single colony on the plate was then used for specific experiments. This concentration is biologically relevant. For example, in Gabriela’s study, biofilm communities were exposed to sub‐MIC levels (1/10, 1/50, and 1/100 of the MIC) of antibiotics for 8 weeks to explore their effects on aquatic environments [15]. Similarly, in Soheir’s study, sub‐MICs (1/2‐1/256 of the MIC) of cefepime were used to investigate the effect of cefepime at sub‐MICs on the biofilm formation *in vitro* [16]. Therefore, it is reasonable to use a sub‐MIC of Gm (30 μg/mL) to observe the adaptive changes of *E. coli* SM10λpir (pUCP24T) under continuous treatment for a period of time.

An increased transfer frequency of the plasmid pUCP24T in *E. coli* SM10λpir was observed. It has been reported that tetracyclines can facilitate the conjugative transfer of RP4, and several antimicrobials, including chlorhexidine, triclosan, Gm, and sulfamethoxazole, at low concentrations can promote HGT of antibiotic resistance [17, 18]. In our research, *E. coli* SM10λpir (pUCP24T)‐E exhibited enhanced HGT capability during adaptation to sub‐MIC Gm. Clinically, during therapy, bacteria are unavoidably exposed to sub‐MIC antibiotic concentrations. To prevent the emergence of strains with higher HGT capacity, it is crucial to manage the duration of drug administration to control the spread of antibiotic resistance. The kor regulon of plasmids uses the *korA*, *korB*, and *korC* repressors to regulate the expression of genes for replication, conjugation, segregation, and host range [19]. The transfer of the conjugative F‐plasmid is mediated by the transfer (tra) region, which encodes nearly 40 genes, 25 of which are essential for this process in *E*. *coli* [20]. The *traJ* is essential for the transcription of the tra operon, and *traI* is one of the key components involved in the initiation and termination of HGT by bacterial conjugation [21]. In our study, the expression of *traI* in *E. coli* SM10λpir (pUCP24T)‐E was upregulated. The *traI* protein catalyzes a site‐specific and strand‐specific cleavage‐joining reaction on form I DNA or single‐stranded DNA. The upregulation of *traI* could strengthen the catalytic action, which may result in the enhanced conjugation frequency between *E. coli* SM10λpir (pUCP24T)‐E and PAO1 during the adaptation. Plasmids with greater copy numbers can confer increased conjugation frequency in the presence of susceptible hosts [22]. In addition to the upregulation of *traI*, a higher plasmid copy number of pUCP24T was observed in *E. coli* SM10λpir (pUCP24T)‐E, which may also lead to an increase in conjugation frequency. This phenomenon aligns with findings in related studies. Mo et al. similarly observed an increased plasmid copy number in *M. smegmatis* correlating with enhanced plasmid transfer to mammalian cells [23]. The study by Guoxiu Xiang reported that strain ECNX52 displayed a higher conjugation frequency, which was attributed to an increase in plasmid copy number when subjected to a continuous pressure of 4 μg/mL meropenem (1/16 of the MIC) [24].

The concept of fitness cost refers to the negative pleiotropic effects on an organism’s fitness in its original environment due to adaptations to a new environment [25]. This concept plays a crucial role in adaptation. Under pressure, the fitness advantage of a resistance trait is likely to exceed any fitness cost. The relative fitness of an offspring strain compared to the original strain will determine the trajectory of genotypes in the absence of selection [26]. In our study, pUCP24T exhibited significant instability after 50 transfers, with a more rapid decline in stability. Tanita Wein’s study also found that antibiotics can lead to plasmid amplification and result in plasmid instability in *E. coli*, showing that positive selection for a plasmid‐encoded gene interferes with the adaptation of plasmid stability [27]. Furthermore, the *E. coli* SM10λpir (pUCP24T)‐E exhibited a lower growth rate and competitive inferiority during adaptation. The adaptive changes under sub‐MIC Gm after 50 transfers imposed a greater metabolic burden on the host.

SNP variation is an important marker that confers an adaptive advantage for bacterial pathogens [28]. In our study, a few SNPs were identified in *E. coli* SM10λpir (pUCP24T)‐E, which may represent one method by which the strains adapt to the constant sub‐MIC Gm treatment. *K. pneumoniae* can enhance its pathogenicity by adopting two opposing infection programs facilitated by easily acquired gain‐of‐function and loss‐of‐function mutations [29]. By analyzing 122 *X. fastidiosa* subsp. *fastidiosa* isolates associated with PD in California, researchers found that 18 nonsynonymous polymorphisms under selective pressures correlated with the adaptation of *X. fastidiosa* to grapevines in California [30]. Further exploration is needed to understand how these SNPs might affect the host’s adaptation in our study.

In addition to multiple SNPs, a total of 1294 DEGs were involved in the adaptation process, with *hycB*, *hycD*, *nikE*, *nikC*, *cspA*, and *nanA* showing relatively higher upregulation, and *gadB*, *gadC*, *yeiQ*, *yjiH*, transcription factors (*appY*, *gadE*), sRNA *arrS*, and sRNA *isrC* showing relatively higher downregulation. The subunits *hycB* and *hycD* belong to the formate hydrogenlyase subunit system, while *nikE* and *nikC* are subunits of ATP‐binding cassette transporters. The genes *gadB* and *gadC* are part of the glutamate decarboxylase (GAD) gene system, and *YeiQ* is related to a putative oxidoreductase, all of which are associated with bacterial metabolism [31]. The *CspA* family in *E*. *coli* consists of cold shock proteins responsible for stress adaptation, and *NanA* is involved in sialic acid metabolism [32]. Bacterial sRNA is a crucial mechanism for regulating bacterial fitness. sRNA *arrS* has been reported to activate *cfa* (cyclopropane fatty acid synthase) post‐transcriptionally by masking an RNase E cleavage site in the *cfa* mRNA 5′ untranslated region, playing a role in the bacterial acid stress response [33]. In *E. coli* SM10λpir (pUCP24T)‐E, the expression of *gadE*, a transcription factor that regulates the glutamate‐dependent acid resistance system 2, was downregulated, and sRNA *arrS*, which positively regulates *gadE* expression post‐transcriptionally, was also downregulated [34]. The mechanisms by which these genes regulate the adaptation of *E. coli* SM10λpir (pUCP24T) under the constant treatment with sub‐MIC Gm will be studied further in our future work. Meanwhile, the functions of sRNA *isrC* in our study were uncharacterized. The potential target genes of sRNA *isrC* were predicted on the RNA–RNA interaction prediction website IntaRNA (https://rna.informatik.uni-freiburg.de/IntaRNA/), and it was suggested that sRNA *isrC* might recognize cytochrome bo oxidase subunit *cyoD*. In *E. coli* SM10λpir (pUCP24T)‐E, sRNA *isrC* was downregulated, while cytochrome bo oxidase (*cyoABCDE*) was upregulated, indicating the possible negative regulation of aerobic respiration enzyme genes by sRNA *isrC*. Thus, we hypothesized that sRNA *isrC* could inhibit aerobic respiration by acting on cytochrome bo oxidase subunit *cyoD*, although this requires experimental confirmation. Compared with fermentation or anaerobic respiration, aerobic respiration is more costly, leading to a heavier fitness cost on the host. In *E. coli* SM10λpir (pUCP24T)‐E, the enhanced expression of key aerobic respiration enzyme genes, including *cyoABCDE* (cytochrome bo oxidase), *atpADGH* (ATP synthase), and *sdhAB* (succinate dehydrogenase), suggested that aerobic respiration was likely potentiated during the adaptation process. The potentially enhanced aerobic respiration may further increase the fitness cost of *E. coli* SM10λpir (pUCP24T)‐E. Furthermore, the functional enrichment analysis by GO and KEGG suggested a possible enhancement of certain physiological processes and metabolic pathways in *E. coli* SM10λpir (pUCP24T)‐E, meaning the metabolic efficiency was likely improved. Moreover, according to GO and KEGG functional enrichment analysis, several physiological processes and metabolic pathways in *E. coli* SM10λpir (pUCP24T)‐E appeared to be strengthened, which is also suggestive of an improvement in metabolic efficiency. Meanwhile, through an integrated approach of mathematical modeling and experiments, it was also found in Sai Varun Aduru’s research that subinhibitory antibiotic treatment results in selection for enhanced metabolic efficiency [35].

Several limitations should be acknowledged in our study. First, the experiment was based on one lineage that had undergone 50 consecutive transfers. Consequently, we cannot determine whether the observed adaptive changes are due to stochastic mutations or a lineage effect. Future studies employing multiple independent lines are necessary to identify which of the reported changes represent convergent, and thus likely adaptive, evolution to the selective pressure. Second, though our data reveal a strong association between enhanced HGT ability and increased plasmid copy number, as well as enhanced *traI* expression, further genetic and functional experiments are needed to confirm the causal relationship. Lastly, the hypothesis that aerobic respiration was reinforced as an adaptive change was based on transcriptional data alone, without supporting physiological or metabolic evidence. Further experiments assessing relevant metabolic indices will be conducted to validate this hypothesis.

## 5. Conclusions

In summary, our study investigated the adaptations of *E. coli* SM10λpir (pUCP24T) under constant treatment with sub‐MIC Gm. During the adaptation process, enhanced conjugation frequency was observed, which may be related to the increased expression of transfer gene *traI* and plasmid pUCP24T copy number. The plasmid pUCP24T instability, lower growth rate of *E. coli* SM10λpir (pUCP24T)‐E, and its competitive inferiority suggested a heavier fitness cost was imposed on the host during adaptation. The enhanced expression of key aerobic respiration enzyme genes suggested that aerobic respiration was likely potentiated, while GO and KEGG functional enrichment analysis might indicate an improvement in metabolic efficiency. Moreover, it is hypothesized that sRNA *isrC* could inhibit aerobic respiration by acting on cytochrome bo oxidase subunit *cyoD*. Further genetic experiments and assessments of relevant metabolic indices are needed to confirm our speculations, revealing the exact mechanisms behind the adaptation of *E. coli* SM10λpir (pUCP24T) under constant sub‐MIC gentamicin treatment.

## Author Contributions

Yuting He: data curation, formal analysis, software and writing–original draft; Guoxiu Xiang: data curation, formal analysis and methodology; Guosheng Zhong: formal analysis, investigation, resources and software; Jianming Zeng: methodology, software, supervision, and validation; Cha Chen: conceptualization, supervision, validation, visualization, and writing–review and editing. Bin Huang: funding acquisition, project administration, supervision, and writing–review and editing.

## Funding

No funding was obtained for this study.

## Ethics Statement

The authors have nothing to report.

## Consent

The authors have nothing to report.

## Conflicts of Interest

The authors declare no conflicts of interest.

## Supporting Information

Additional supporting information can be found online in the Supporting Information section.

## Supporting information


**Supporting Information 1** Supporting Table 1: The primers used in conjugative transfer gene expression.

**Supporting Information 2** Supporting Table 2: The predicted SNPs that differed in the *E. coli* SM10λpir (pUCP24T) and *E. coli* SM10λpir (pUCP24T)‐E.

## Data Availability

The data that support the findings of this study are openly available in NCBI database at https://www.ncbi.nlm.nih.gov/.
