# Supplementary material for: The Adaptations of E. coli SM10λpir (pUCP24T) Under Constant Sub‐MIC Gentamicin Treatment
Source: Can J Infect Dis Med Microbiol. 2026 May 12;2026:6978370. doi: 10.1155/cjid/6978370 (PMC13168525; doi:10.1155/cjid/6978370)
Supplement: Supplementary file 1 — Supporting Information 1 Supporting Table 1: The primers used in conjugative transfer gene expression. [file CJID-2026-6978370-s001.docx]

| Primers | Sequence (5’-3’) |
| --- | --- |
| *KorA-F* | GCTTACCGAAAGCCAGTTCCAG |
| *KorA-R* | GCAAGTTCTTGTCCTCGAACGC |
| *KorB-F* | AAGGAAAAGGGCGCGAAGGAG |
| *KorB-R* | TCGATGAGCGCGACCAGTTTC |
| traI-F | ATCACGAAGGGAACCATCATC |
| traI-R | TTGAACTCTGCTGTGCCGTTGAC |
| traJ-F | CGAACGAAGAGCGATTGAGG |
| traJ-R | TCGTCGGTGAGCCAGAGTTT |
| rpoD-F | TATCTGCTGGAACAGTACGATCGTG |
| rpoD-R | TGTTGTCATCATCGGCGCTG |

**Table S1 The primers used in conjugative transfer gene expression**
