# Supplementary material for: The Adaptations of E. coli SM10λpir (pUCP24T) Under Constant Sub‐MIC Gentamicin Treatment
Source: Can J Infect Dis Med Microbiol. 2026 May 12;2026:6978370. doi: 10.1155/cjid/6978370 (PMC13168525; doi:10.1155/cjid/6978370)
Supplement: Supplementary file 2 — Supporting Information 2 Supporting Table 2: The predicted SNPs that differed in the E. coli SM10λpir (pUCP24T) and E. coli SM10λpir (pUCP24T)‐E. [file CJID-2026-6978370-s002.docx]

| **Table S2 The predicted SNPs that differed in the *E. coli* SM10λpir (pUCP24T) and *E. coli* SM10λpir (pUCP24T)-E** | | | | | |
| --- | --- | --- | --- | --- | --- |
| Position | *E. coli* SM10λpir (pUCP24T) | *E. coli*  SM10λpir (pUCP24T)-E | Annotation | Gene | Description |
| pUCP24T | A | G | intergenic (+359/+2024) | *rep* → / ← *aacC1* | replication protein/gentamycin acetyltransferase‑3‑1 |
|  | A | C | intergenic (+1511/+872) | *rep* → / ← *aacC1* | replication protein/gentamycin acetyltransferase‑3‑1 |
| *E. coli* SM10λpir | T | C | L375M (TTA→CTG) | *icd* → | e14 prophage; isocitrate dehydrogenase, specific for NADP+ |
|  | A | G | L375M (TTA→CTG) | *icd* → | e14 prophage; isocitrate dehydrogenase, specific for NADP+ |
|  | G | A | L80L (TTG→TTA) | *pinE* → | e14 prophage; site‑specific DNA recombinase |
|  | C | A | R81R (CGC→CGA) | *pinE* → | e14 prophage; site‑specific DNA recombinase |
|  | A | G | E82E (GAA→GAG) | *pinE* → | e14 prophage; site‑specific DNA recombinase |
|  | C | G | G84G (GGC→GGG) | *pinE* → | e14 prophage; site‑specific DNA recombinase |
|  | C | T | I85I (ATC→ATT) | *pinE* → | e14 prophage; site‑specific DNA recombinase |
|  | C | T | N86N (AAC→AAT) | *pinE* → | e14 prophage; site‑specific DNA recombinase |
|  | T | C | R88R (CGT→CGC) | *pinE* → | e14 prophage; site‑specific DNA recombinase |
|  | G | T | L90L (CTG→CTT) | *pinE* → | e14 prophage; site‑specific DNA recombinase |
|  | G | T | T91T (ACG→ACT) | *pinE* → | e14 prophage; site‑specific DNA recombinase |
|  | T | C | D92D (GAT→GAC) | *pinE* → | e14 prophage; site‑specific DNA recombinase |
|  | T | A | S93S (TCA→AGT) | *pinE* → | e14 prophage; site‑specific DNA recombinase |
|  | C | G | S93S (TCA→AGT) | *pinE* → | e14 prophage; site‑specific DNA recombinase |
|  | A | T | S93S (TCA→AGT) | *pinE* → | e14 prophage; site‑specific DNA recombinase |
|  | C | G | T96T (ACC→ACG) | *pinE* → | e14 prophage; site‑specific DNA recombinase |
|  | A | T | S97S (AGC→TCA) | *pinE* → | e14 prophage; site‑specific DNA recombinase |
|  | G | C | S97S (AGC→TCA) | *pinE* → | e14 prophage; site‑specific DNA recombinase |
|  | C | A | S97S (AGC→TCA) | *pinE* → | e14 prophage; site‑specific DNA recombinase |
|  | A | T | T98S (ACA→TCT) | *pinE* → | e14 prophage; site‑specific DNA recombinase |
|  | A | T | T98T (ACA→ACT) | *pinE* → | e14 prophage; site‑specific DNA recombinase |
|  | A | G | G101G (GGA→GGG) | *pinE* → | e14 prophage; site‑specific DNA recombinase |
|  | C | T | R102R (CGC→CGT) | *pinE* → | e14 prophage; site‑specific DNA recombinase |
|  | T | C | F105F (TTT→TTC) | *pinE* → | e14 prophage; site‑specific DNA recombinase |
|  | T | C | H106H (CAT→CAC) | *pinE* → | e14 prophage; site‑specific DNA recombinase |
|  | G | T | V107V (GTG→GTT) | *pinE* → | e14 prophage; site‑specific DNA recombinase |
|  | T | C | intergenic (‑140/‑228) | *traJ*← / → *traK* | conjugal transfer relaxosome component TraJ/conjugal transfer protein TraK |
